# Supplementary material for: Content-rich biological network constructed by mining PubMed abstracts
Source: BMC Bioinformatics. 2004 Oct 8;5:147. doi: 10.1186/1471-2105-5-147 (PMC528731; doi:10.1186/1471-2105-5-147)
Supplement: Additional File 2 — The original results of the above study (non-essential files are deleted to keep the file size under the limit set by BMC bioinformatics). [file 1471-2105-5-147-S2.bz2 › chilibotAdditionalFile2/dip40/141ID7739537E501/html/TFDP1_RB1.html]

 


 **TFDP1** and **RB1** 
  
Found 66 abstracts in PubMed, retrieved 40.  
 

 What does Google say? 
 PDF only 
| .edu only 

---

**Interactive relationship** (e.g. stimulation, inhibition, etc)

**Inhibitory relationship**- Co expression of the retinoblastoma  [ **RB1** ]  gene product RB abolishes baseline E2F 1 DP 1  [ **TFDP1** ]  transcriptional activity as well as TRIP Br KRIP 1 co activation, both of which are restored by the adenovirus E1A oncoprotein.  Ref: 11331592 EMBO J, 2001
- Although the mdm2 gene is thought to act as an oncogene by blocking the activity of p53, recent studies indicate that mdm2 can act independently of p53 and block the G1 cell cycle arrest mediated by members of the retinoblastoma  [ **RB1** ]  gene family and can activate E2F1 DP1  [ **TFDP1** ]  and the cyclin A gene promoter.  Ref: 9858609 Mol Cell Biol, 1999
- The retinoblastoma  [ **RB1** ]  tumor suppressor protein has been shown to induce growth arrest by binding to E2F TFDP  [ **TFDP1** ]  and repressing its activity.  Ref: 9027491 Genomics, 1997
- The transcription factor DRTF1  [ **TFDP1** ]  E2F is implicated in the control of cellular proliferation due to its interaction with key regulators of cell cycle progression, such as the retinoblastoma  [ **RB1** ]  tumour suppressor gene product and related pocket proteins, cyclins and cyclin dependent kinases.  Ref: 7542760 Oncogene, 1995
- The cellular transcription factor DRTF1  [ **TFDP1** ]  E2F is implicated in the control of early cell cycle progression due to its interaction with important regulators of cellular proliferation, such as pocket proteins for example, the retinoblastoma  [ **RB1** ]  tumour suppressor gene product, cyclins and cyclin dependent kinase subunits.  Ref: 8537434 J Cell Sci, 1995
- DRTF1  [ **TFDP1** ]  E2F is believed to coordinate and integrate the transcription of cell cycle regulating genes, for example, those involved in DNA synthesis, with the activity of regulatory proteins, such as the retinoblastoma  [ **RB1** ]  tumor suppressor gene product pRb, which modulate its transcriptional activity.  Ref: 8816502 Mol Cell Biol, 1996
**Neutral relationship**- During most of the G1 phase, E2F1 DP1  [ **TFDP1** ]  activity is repressed by the retinoblastoma  [ **RB1** ]  gene product RB, which directly contacts the E2F1 activation domain and silences it.  Ref: 8932363 Nucleic Acids Res, 1996
- Recently, Mdm2 has been found to bind several other proteins that function to regulate cell cycle progression, including the E2F 1 DP1  [ **TFDP1** ]  transcription factor complex and the retinoblastoma  [ **RB1** ]  tumor suppressor protein.  Ref: 8943068 Proc Natl Acad Sci U S A, 1996
- The cellular transcription factor DRTF1  [ **TFDP1** ]  E2F is implicated in this integration process by virtue of its physical interaction and control by key regulators of proliferation, such as retinoblastoma  [ **RB1** ]  protein, cyclins and cyclin dependent kinases and regulation of target genes required for cell cycle progression.  Ref: 7478568 Oncogene, 1995
- E2F DP1  [ **TFDP1** ]  heterodimers bind more efficiently than homodimers to DNA, retinoblastoma  [ **RB1** ]  gene product as well as the adenovirus E4 protein and DP1  [ **TFDP1** ]  can stimulate transcription from E2F sites in a synergistic fashion.  Ref: 7784053 Oncogene, 1995

**Non-interactive relationship** (e.g. studied together, co-existance, homology, etc.)

- Because Cdk4 6 phosphorylates retinoblastoma  [ **RB1** ]  protein pRb family members that then modulate the transcriptional activity of E2F DP1  [ **TFDP1** ]  complexes, we examined the involvement of these components in DNA damage evoked neuronal death.  Ref: 10777774 J Neurosci, 2000
- Release of E2F1 DP1  [ **TFDP1** ]  heterodimers from repression mediated by the retinoblastoma  [ **RB1** ]  tumor suppressor pRB triggers cell cycle entry into S phase, suggesting that E2F1 and DP1  [ **TFDP1** ]  proteins must act in unison, either to facilitate or to suppress cell cycle progression.  Ref: 12588846 Development, 2003
- Phosphorylation of the retinoblastoma  [ **RB1** ]  protein is also increased following induction of E2F 1 DP 1  [ **TFDP1** ] , suggesting that E2F can feed back on the retinoblastoma  [ **RB1** ]  protein, presumably through activation of cyclin A and or E associated kinase activity.  Ref: 8780882 Cell Growth Differ, 1996
- E2F1, the first cloned member of this family, is regulated during the cell cycle at the mRNA level by changes in transcription of the E2F1 gene and at the protein level by complex formation with proteins such as the retinoblastoma  [ **RB1** ]  gene product pRB, cyclin A and DP1  [ **TFDP1** ] .  Ref: 8964493 Gene, 1996
- mdm2 has recently been shown to interact with the retinoblastoma  [ **RB1** ]  tumor suppressor protein p Rb, and the E2F 1 and DP1  [ **TFDP1** ]  transcription factors.  Ref: 9322885 Leuk Lymphoma, 1997
- We have shown that p202, an IFN inducible 52kDa primarily nuclear phosphoprotein whose expression in transfected cells inhibits cell proliferation, interacts with the retinoblastoma  [ **RB1** ]  tumor suppressor protein pRb and the transcription factor E2F E2F 1 DP 1  [ **TFDP1** ]  in vitro and in vivo.  Ref: 9233764 Oncogene, 1997
